# **Recent developments in carbon-based two-dimensional materials: synthesis and modification aspects for electrochemical sensors**

**DOI:** 10.1007/s00604-020-04415-3

**Published:** 2020-07-12

**Authors:** Eva-Maria Kirchner, Thomas Hirsch

**Affiliations:** Institute of Analytical Chemistry, Chemo- and Biosensors, University of Regensburg, 93040 Regensburg, Germany

**Keywords:** Graphene, Reduced graphene oxide, Carbon nanomaterial, Exfoliation, Electrochemical sensor

## Abstract

This review (162 references) focuses on two-dimensional carbon materials, which include graphene as well as its allotropes varying in size, number of layers, and defects, for their application in electrochemical sensors. Many preparation methods are known to yield two-dimensional carbon materials which are often simply addressed as graphene, but which show huge variations in their physical and chemical properties and therefore on their sensing performance. The first section briefly reviews the most promising as well as the latest achievements in graphene synthesis based on growth and delamination techniques, such as chemical vapor deposition, liquid phase exfoliation via sonication or mechanical forces, as well as oxidative procedures ranging from chemical to electrochemical exfoliation. Two-dimensional carbon materials are highly attractive to be integrated in a wide field of sensing applications. Here, graphene is examined as recognition layer in electrochemical sensors like field-effect transistors, chemiresistors, impedance-based devices as well as voltammetric and amperometric sensors. The sensor performance is evaluated from the material’s perspective of view and revealed the impact of structure and defects of the 2D carbon materials in different transducing technologies. It is concluded that the performance of 2D carbon-based sensors is strongly related to the preparation method in combination with the electrical transduction technique. Future perspectives address challenges to transfer 2D carbon-based sensors from the lab to the market.

**Graphical abstract:**
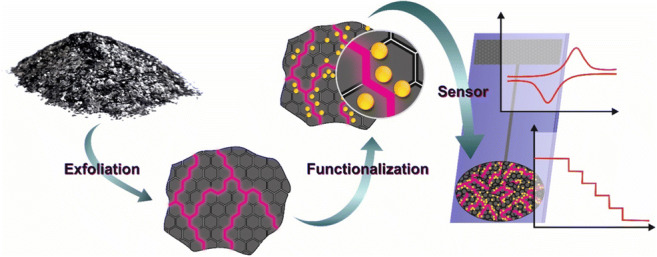
Schematic overview from synthesis and modification of two-dimensional carbon materials to sensor application.

Schematic overview from synthesis and modification of two-dimensional carbon materials to sensor application.

## Introduction

Ten years ago, J. Justin Gooding and Filip Braet have asked, “Should you use nanotubes or graphene as carbon nanomaterial in biosensors?” [1], motivated by numerous publications on carbon nanotubes in biosensing and the start of the graphene hype, which was discovered as a single flake about 15 years ago [2]. One decade ago, only a few papers using 2D carbon materials have been published and research on this topic was in the early stage where all those interesting properties coming with this material have been described; some of them even outperforming the ones known for carbon nanotubes (CNTs). Today, carbon-based nanomaterials have become one of the dominating materials in many sensor applications. A web-of-science survey revealed up to now more than 2.200 publications related to nanomaterial and sensing and roughly about 50% of them deal with carbon nanomaterials. On a closer look, about half of the carbon materials are from the so-called graphene family. These impressive numbers raise the question why 2D carbon nanomaterials got so popular in sensing that one finds almost three times more publications with this class of materials compared to CNTs. Graphene, ideally consisting of a honeycomb structured monolayer of sp^2^-hybridized carbon atoms, is characterized as an almost transparent, chemically inert material, exhibiting high carrier mobility, as well as excellent electric and thermal conductivity [3, 4]. These properties are often mentioned together with a high surface-to-volume ratio as motivation why 2D carbon nanomaterials are used in (bio)analytical applications, especially for sensor development where also costs of materials come into play. In electroanalytical applications, carbon-based electrodes outperform many other materials, such as noble metals, because they are less prone to surface fouling and they are known for a wider electrochemical potential window ranging from − 1.5 to + 1 V vs. Ag/AgCl [5–8].

For sensor development, it is desired that the electrode fabrication can be performed in an easy way, allowing mass production, which is not in coincidence with the preparation of individual graphene flakes from graphite by the famous scotch tape method [2]. Many other manufacturing methods have been developed, and it turned out by sophisticated material characterization processes that those two-dimensional carbon materials are somehow similar to graphene, even when they contain many defects compared to an ideal graphene flake [2]. In the last years, the number of exfoliation protocols to obtain 2D carbon materials has risen, mostly motivated by the goal to produce a material, which comes as close as possible to a perfect graphene but to be accessible in large scale. It became clear, as for every nanomaterial, that intrinsic material properties with regard to the exfoliation process are differing a lot due to their flake morphology, which are varying in the size of the flakes, the amount of defects, the number of layers (mono-, bi-, few-, multi-layers), and the doping ratios [9, 10]. All the structural and chemical variations introduced during the processing of such materials change the intrinsic material properties. This leads to the question of how different exfoliation methods affect the properties of electrochemical sensors based on 2D carbon nanostructures, which is evaluated in this review.

## Synthesis and functionalization

Preparation techniques for 2D materials are often classified either bottom-up or top-down approaches which are capable to create nanomaterials with a varying distribution of lattice defects. These include vacancies, grain boundaries, oxygen functional groups, dangling bonds, and Stone-Wales defects resulting in a certain degree of functionalization. Excellent reviews are published on the high diversity of preparation methods to yield two-dimensional materials [10–14]. With regard to the preparation method, the intrinsic characteristics of 2D materials can be chosen, as well as parameters such as the potential for scale-up-synthesis and processability, which influence their use as sensor material [10, 13, 15–17]. In the following, the most important fabrication techniques and functionalization strategies are critically evaluated.

## Chemical exfoliation

**Fig. 1:**
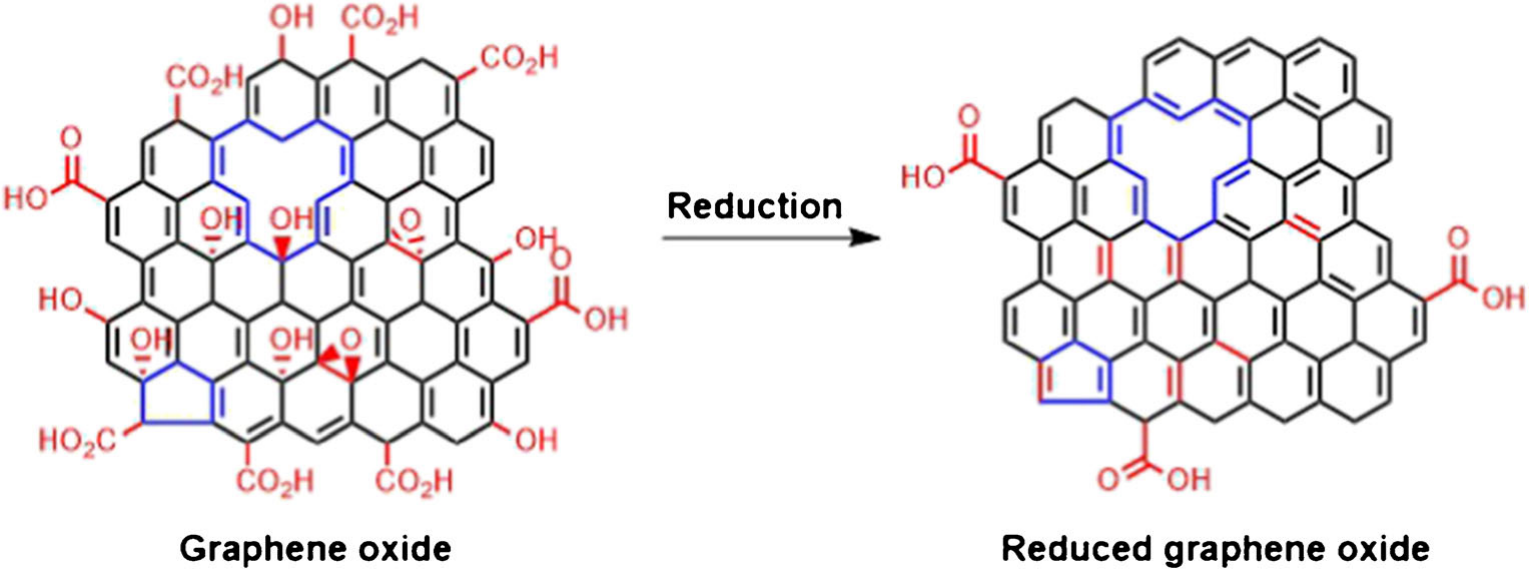
Chemical structure of highly defective graphene oxide. The reduction process leads to a restored sp^2^-hybridized carbon lattice with oxygen moieties at the edges of the flakes. The elimination of structural defects within the material is not feasible

The chemical synthesis is the widest distributed technique to obtain graphene, ensured by the simple instrumentation and the advantage to obtain aqueous dispersions of 2D carbon nanomaterials. Graphene oxide (GO) is synthesized by treating graphite with a strong acid and an oxidizing species, which delaminates the graphite crystal structure introducing oxygen functional groups. This material can be reduced in many ways to reduced graphene oxide (rGO), which is widely applied as sensor layer. Brodie paved the way for the graphene oxide synthesis already in 1859 by oxidizing graphite with potassium chlorate and fuming nitric acid [18], followed by Staudenmayer around 40 years later who modified Brodie’s synthetic route slightly by stepwise oxidation of the graphite in a mixture of sulfuric acid and nitric acid. Potassium chlorate was added in small portions to avoid explosive reactions in a more acidic environment [19]. In 1957 Hummers and Offeman established a safer production method for GO. Graphite was oxidized by the addition of KMnO_4_ and NaNO_3_ in concentrated sulfuric acid [20]. This synthetic route is still applied nowadays by slight modifications with additional amounts of oxidizing agents for an increased oxidation rate of the material [21, 22]. The high degree of oxygen-containing functional groups distributed along the surface of the GO flake leads to an electrically insulating material. The interlayer spacing of the sheets is increased by the oxidation of graphite with oxidizing agents forming epoxy, hydroxy, and carboxyl groups [23]. Oxidative cleavage cause in-plane voids and cracks at the edges [24]. The subsequent reduction of GO eliminates the oxygen residues, restoring the sp^2^-hybridized carbon system and its conductivity. Figure 1 indicates that the reduction step cannot remove structural defects of the material, which originate from the oxidation process [24]. Reduced graphene oxide is classified as highly defective material. The harsh oxidation and subsequent reduction process introduce structural defects as well as oxygen moieties [25].

The thermal treatment is the simplest way to reduce GO [26–28]. Many of the thermal reduction steps to form rGO are performed hydrothermally in an autoclave to reduce the precursor material under high temperature and pressure [22, 29, 30]. A chemical reduction, mainly by hydrazine, ascorbic acid, sodium citrate, hydroiodic acid, or sodium borohydrate is also quite common [29, 31–33].

An advantage of the aqueous dispersed rGO compound is the facile transfer mechanism for chemically synthesized 2D materials [13, 30, 34–36]. Spin-casting requires an ink-formulation to ensure a fast evaporation of the solvent as well as the application of ideal processing parameters in order to form a coherent, homogeneous film [37]. Drop coating from aqueous dispersion seems easier but is challenging in resulting homogeneous films.

Chemical synthesis of 2D materials provides a large degree of freedom to adjust process parameters. So far, these options have not been fully exploited, e.g., by the design of 2D materials with a controlled number of defects. Such materials might be of great interest in sensing applications. It is observed for surface functionalization by nanoparticle (NP) deposition that the structural irregularities act as nucleation sites for the particles [35]. Defects allow the introduction of bandgaps, which might be beneficial in field effect transistors [38].

**Fig. 2:**
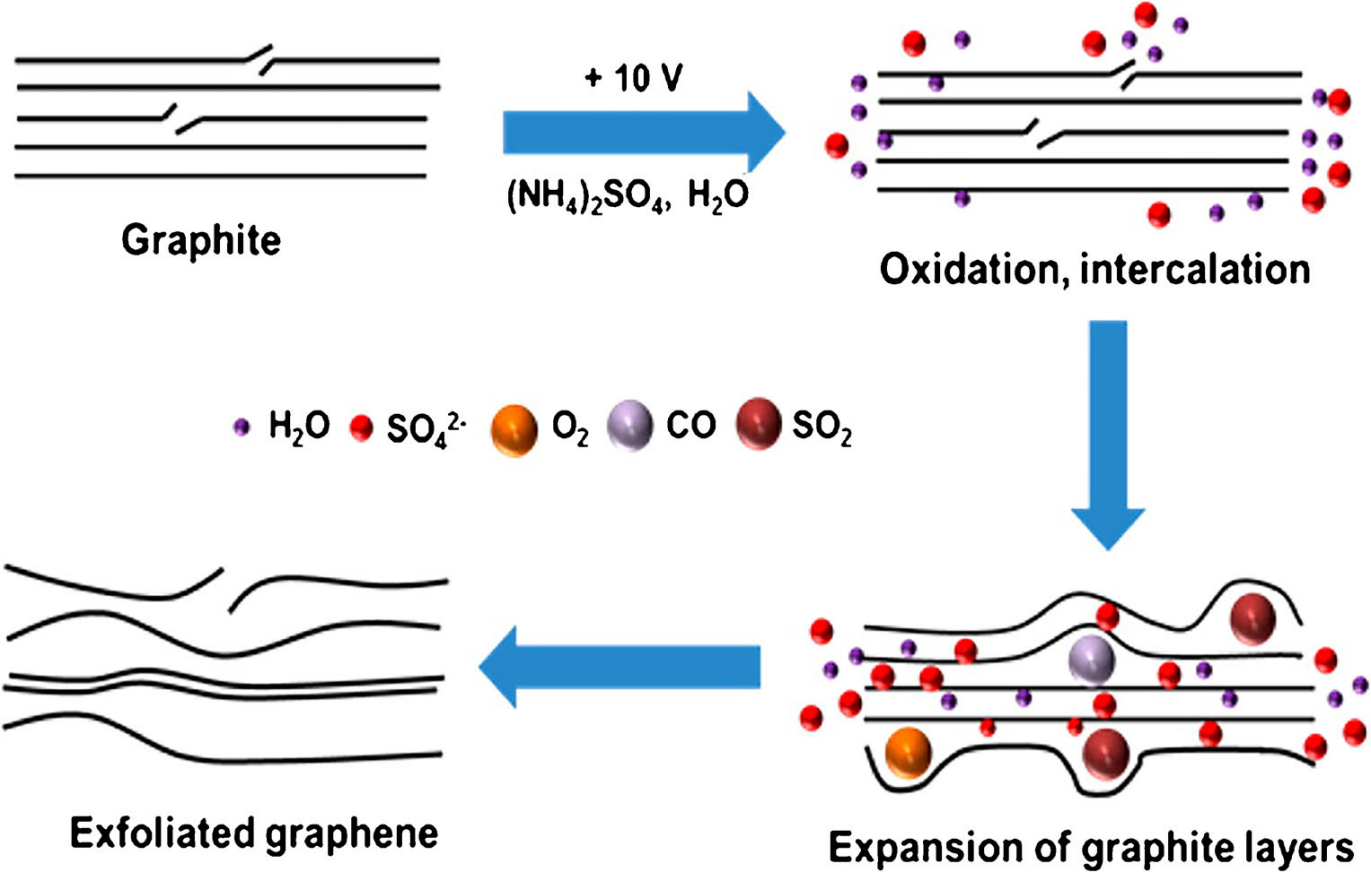
Scheme of an anodic electrochemical exfoliation process of graphite. Oxidation of water causes the formation of oxygen-containing species attacking the edges and grain boundaries of graphite promoting the SO_4_^2−^ intercalation and exfoliation of graphene layers. Reprinted with permission from [44]. Copyright 2014 American Chemical Society

In comparison to chemical synthesis, which needs, in total, several days to proceed, the exfoliation can be speeded up to hours by applying a strong electrical potential to graphite electrodes. Upon application of an electrical voltage, solvated ions of the electrolyte intercalate within the graphite working electrode, weakening the interlayer forces and driving the individual flakes apart, which are released into the electrolyte [39, 40]. The application of a negative potential to the graphite electrode in organic medium force positively charged ions between the layers of the bulk material, which is called cathodic electrochemical exfoliation. The applied voltage, size of the anions, and the type of solvent do not only affect the intercalation process but also have an impact on the quality of processed material. The cathodic electrochemical exfoliation is either carried out by intercalating Li^+^ ions or quaternary ammonium salts in organic solvents [41–43]. Anodic exfoliation is performed in aqueous solution, where anions intercalate between the graphite layers upon electrolysis, triggering the delamination (Fig. 2). Parvez et al. extensively studied the exfoliation efficiency of various inorganic salts (NH_4_Cl, Na_2_SO_4_, (NH_4_)_2_SO_4_, NaNO_3_, K_2_SO_4_, and NaClO_4_). Sulfate yields large graphene flakes with a lateral size up to 44 μm (> 85% with ≤ 3 layers) and no exfoliation was observed for ClO_4_^−^, Cl^−^, and NO_3_^−^ [44].

Raman spectroscopy, a versatile tool for non-destructive optical characterization of 2D materials, revealed that the material got oxidized during the exfoliation procedure exhibiting major structural defects [45]. The electrochemical exfoliation procedure is not that widely integrated into sensor preparation so far. A reason might be that this method was only recently developed. Several advantages such as less labor-intense, easier control of flake size, and defects by the ideal choice of intercalating salts and electrochemical potentials let one expect that electrochemical exfoliation might become more attractive in the future.

## Chemical vapor deposition

**Fig. 3:**
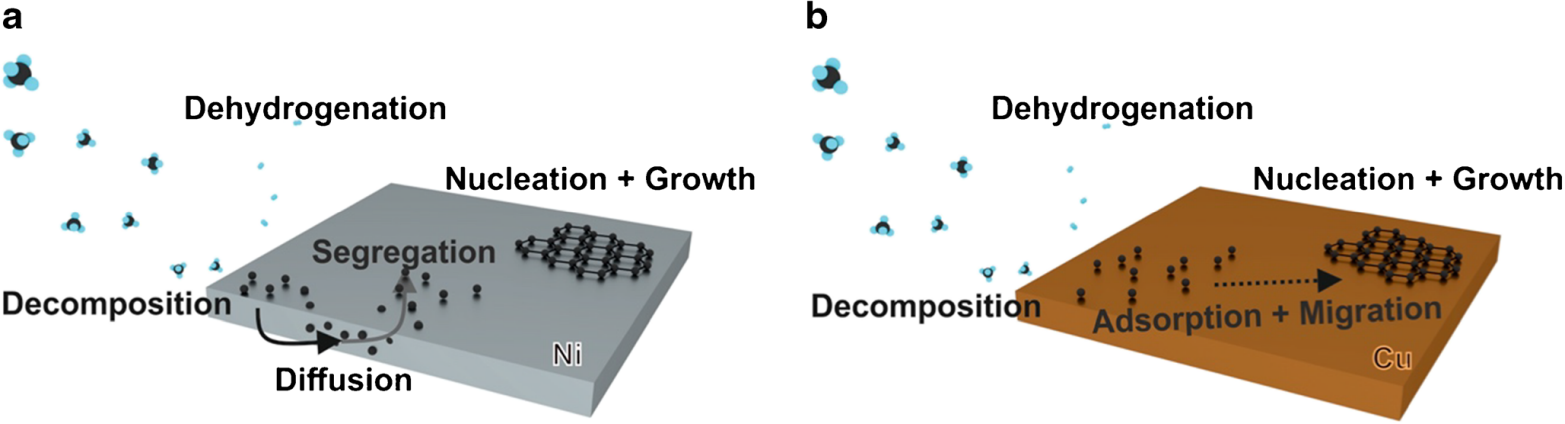
Schematic illustration of the cvdG growth process on a carbon-soluble metal support (Ni) where the hydrocarbon molecules, e.g., methane, decompose and the C atoms diffuse into the bulk of the metal. The C atoms segregate upon supersaturation, nucleate, and grow a carbon layer (**a**). On a Cu substrate, the hydrocarbons decompose as carbon poorly segregates in copper. The adsorbed adatoms migrate along the surface, nucleate, and form the graphene layer (**b**). As depicted in detail by Muñoz and Gómez-Aleixandre [52]

Chemical vapor deposition (CVD) gained its popularity due to the possibility to grow large high-quality 2D carbon materials [46]. The growth mechanism of this bottom-up technique depends on the metallic catalyst triggering the layer deposition. There are two prominent representatives of catalytic support, Ni and Cu, which differ in the deposition mechanism as highlighted in Fig. 3. Generally, the growth of graphene on Ni is hardly controllable. The high solubility of C atoms in Ni can lead to the formation of CVD graphene (cvdG) domains with different thickness resulting in an inhomogeneous layer. In contrast to Ni, the growth on Cu bases on a self-limiting surface adsorption process, attributed to the low solubility of C atoms in Cu [47–49]. The decomposed carbon atom nucleates on top of the metallic surface forming ordered crystals subsequently growing a two-dimensional carbon layer [39, 49, 50]. The issue of multi-layered cvdG grown on Ni seemed to be solved by cvdG on Cu substrates on the price of grain boundaries and wrinkles along its surface [49–51], which might affect the physical properties of the material and therefore the sensing performance.

Apart from the deposition, the layer transfer to a substrate of choice is still challenging for 2D carbon materials prepared by CVD. Etching of the metallic substrate increases the cost and introduces ionic impurities [49, 53]. A post-introduction of defects can be caused by electrochemical or mechanical delamination [54]. The films are prone to the introduction of cracks and wrinkles, which lead to a deterioration of the physical properties [55]. In contrast, a systematic introduction of defects by transferring cvdG, specifically to form a wrinkled layer, was found to induce a bandgap [56]. Besides the transfer, also a modification might induce defects within the CVD grown layer. To establish an NO_2_ senor, a pulsed laser deposition of ZrO_2_NPs- or AgNPs on cvdG did not only decorate the carbon layer but also introduce defects [57].

**Fig. 4:**
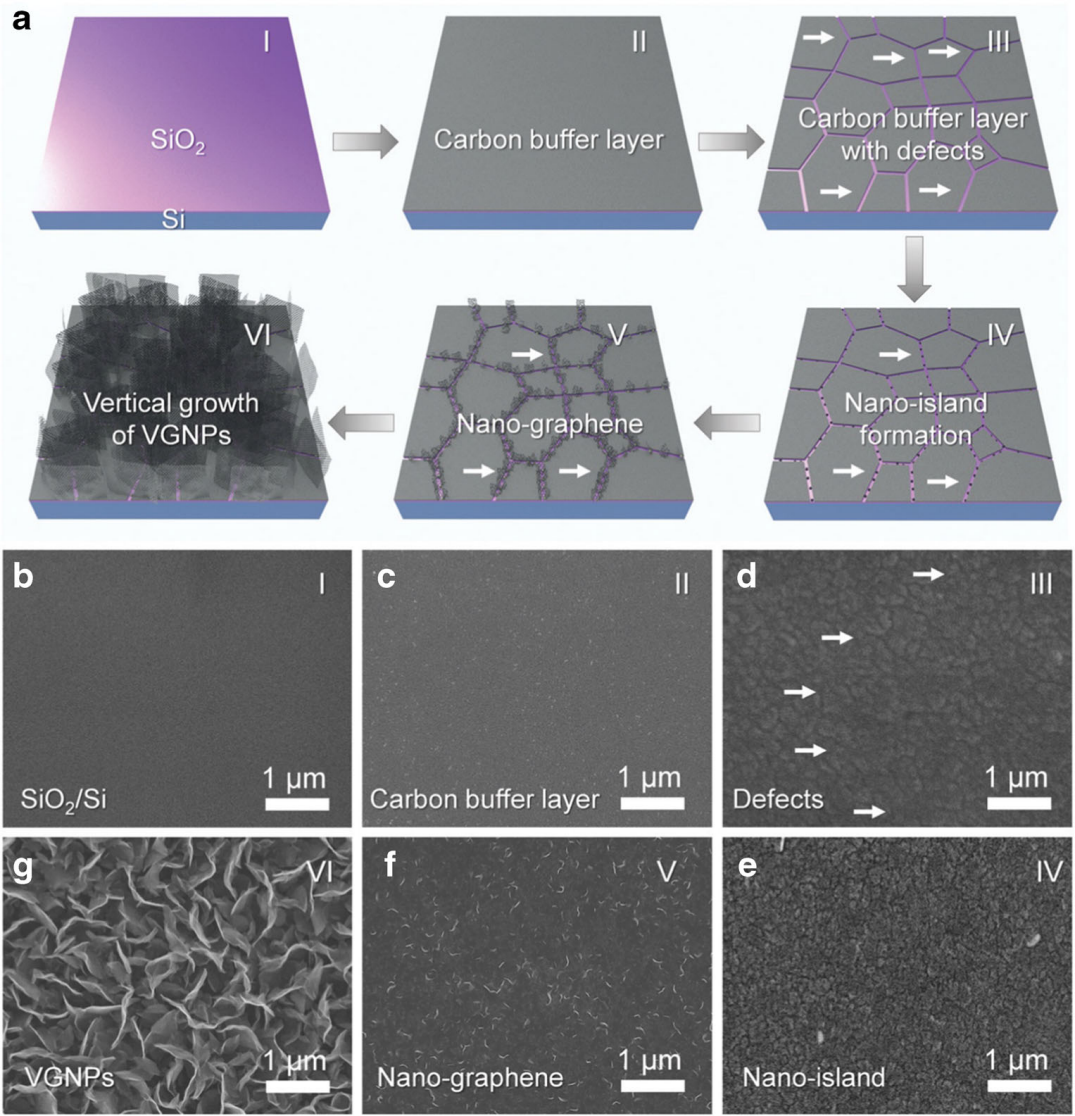
Illustration of the preparation process of graphene derived by plasma-assisted CVD growth (**a**). The numbers (I–IV) scheme the corresponding SEM images (**b**–**g**). The SiO_2_/Si support (I, **b**) was modified by depositing a carbon buffer layer (II, **c**), followed by the introduction of defects within the material (III, **d**) forming nanoislands (IV, **e**). At the edges of the nanoislands, the growth of graphene is initiated (V, **f**), resulting in the vertical growth of graphene flakes (VI, **g**). Reprinted from [58] with permission from The Royal Society of Chemistry

Even though, research aims for ongoing improvement of the growth mechanism to achieve large-area films of high-qualitative graphene material, mostly for optoelectronic applications such as displays, it became apparent that there is no need for the growth of large films in sensing applications, since most electrochemical sensors operate with small electrodes in the mm^2^ range or even smaller. Moreover, the preparation method still is time-consuming and compensates a lot of energy due to the high operating temperature of around 1000 °C. To circumvent the need of a metallic catalyst requiring a subsequent transfer step as well as to reduce the deposition temperature, the growth of graphene on non-conductive materials, such as SiO_2_, was developed [58]. Figure 4 schemes the growth procedure, which does not result in a horizontal growth of the carbon material, but deposits vertically oriented graphene flakes, which additionally enhances the surface area of the carbon layer. In terms of larger surface-to-volume ratio, such vertically aligned carbon 2D materials should outperform horizontally aligned carbon layers in sensor applications which rely on a large specific surface area.

## Liquid phase exfoliation

**Fig. 5:**
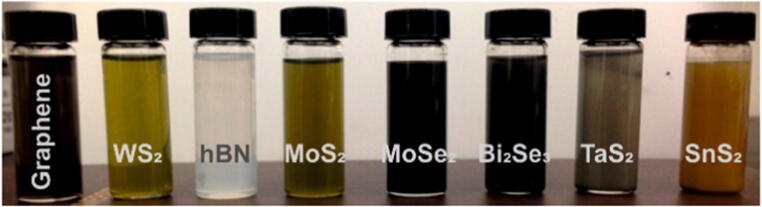
Variety of liquid-phase exfoliated 2D materials dispersed in appropriate solvent. Reprinted with permission from [59]. Copyright 2015 American Chemical Society

An alternative synthesis routine was investigated to obtain high qualitative 2D materials dispersed in liquid medium. This method can be applied to numerous bulk crystals, yielding colloidal stable dispersions (Fig. 5), which are supposed to facilitate the processability of the material and a scale-up in liter-sized batches is possible.

In contrast to chemical synthesis, either by oxidizing graphite under harsh conditions yielding GO, the liquid phase exfoliation (LPE) takes advantage of a direct, mild exfoliation of the bulk material in liquid medium [60, 61]. It yields materials free of defects within the basal plane. This method is solely based on the application of ultrasound or shear forces and does not need further sophisticated equipment. Liquid phase exfoliation imposes three steps. First, the weak interlayer van der Waals forces must be overcome by introducing energy during the exfoliation procedure. Second, the exfoliated nanosheets have to be stabilized against reaggregation by choosing an appropriate solvent or surfactant. Third, the purification and size selection of the obtained batch of exfoliated layered material. The last step is necessary to get rid of unexfoliated material as well as to distinguish fractions with a certain size and number of layers [62].

**Fig. 6:**
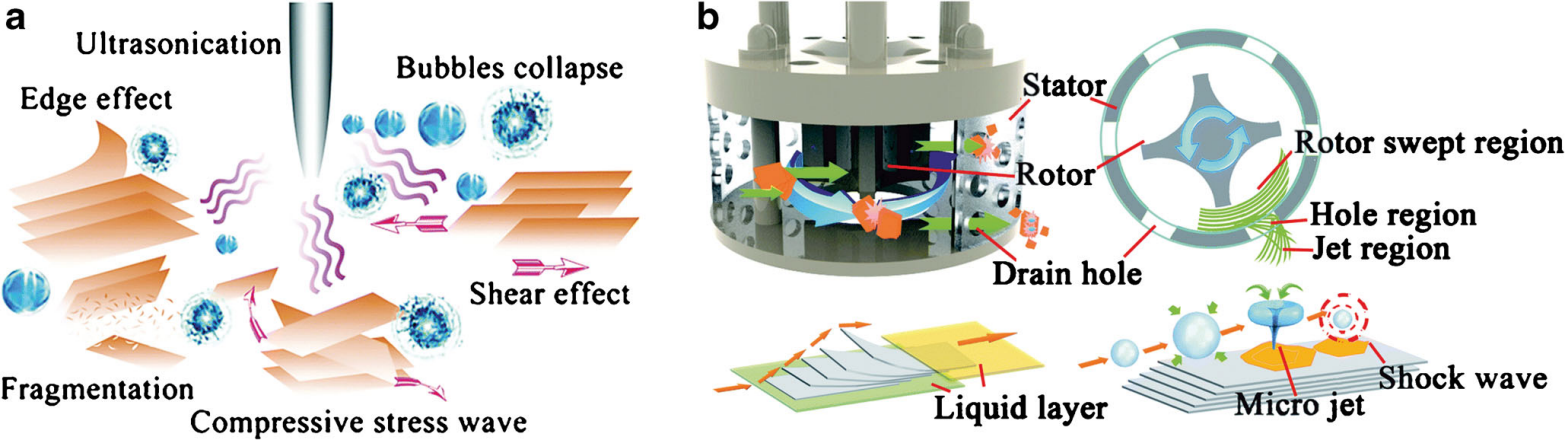
Scheme of liquid phase exfoliation strategies based on ultrasound (**a**) [64] or shear forces (**b**) [65]. Reproduced from [64] with permission from the PCCP Owner Societies. Adapted from [65] with permission from The Royal Society of Chemistry

Coleman et al. pioneered the field of LPE [63]. The exfoliation bases on sonication, which induce the formation of cavitation, generates micro jets and shock waves (Fig. 6). The resulting tensile stress and shear stress cause the delamination and fragmentation of the bulk material [12, 64, 65]. It is reported that the material preparation based on shear forces induced by rotating blade mixers is more applicable in industrial-scale production compared to ultrasound [66]. The exfoliation time for sonication requires a few hours, whereas shear exfoliation consumes more time to yield the same amount of 2D material. Nevertheless, larger volumes in liter range can be produced by shear exfoliation contrary to ultrasound, which yields fractions of less than 0.5 L. [62]

The choice of the solvent is the key in terms of production. Apart from the effective delamination of the material, it is mandatory to keep the dispersion colloidal stable. This can be achieved when the solvent and delaminated material match in surface energies and possess similar Hansen solubility parameters [67, 68]. High boiling point solvents like *N*,*N*-dimethylformamide (DMF) and *N*-methyl-1-pyrrolidone (NMP) were found to be versatile dispersants [69–71]. One might assume that the simple LPE exfoliation is an excellent method to produce almost defect-free 2D materials without any additional impurities, but this comes for the price of delaminated flakes with inhomogeneous morphology. A reproducible preparation of defined mono- to multi-layered flakes with varying flake size is challenging.

Liquid-phase exfoliation is gaining more interest due to the timesaving, up-scalable exfoliation, which yield materials mostly unchanged in their chemical structure ranging from dispersed monolayers to few-layers [72]. So far, only a very limited number of sensor application make use of 2D materials prepared by LPE. One reason might be that the solvents which warrant colloidal stability are hardly removed by washing steps or thermal treatment [73], which may cause a deterioration of electronics and sensing devices.

In contrast to many noble metal, 2D carbon materials have been reported as stable against electrode fouling. This was demonstrated by copper electrodes which were partly covered by cvdG. Even for inner sphere redox couples, no apparent fouling was observed [74].

## Functionalization

The high surface-to-volume ratio and large sp^2^-hybridized carbon system are key properties of 2D carbon materials to be integrated into a sensor device. Analyte interaction can take place via π-interaction of the extended aromatic carbon system or by hydrogen bonding with the oxygen residues present within and at the edge-planes of the graphene flakes. The introduction of additional recognition elements is highly desired to enhance the sensitivity and selectivity of the carbon material. Georgakilas et al. published detailed reviews on the functionalization of graphene, covalently and non-covalently [75, 76]. Therefore, the most common modification routines are only briefly introduced, with emphasis on their use in electrochemical sensors. Non-covalent modifications take place via π-stacking, van der Waals forces, ionic interactions as well as due to hydrogen bonds [76]. These functionalization strategies are less selective, but the sp^2^-hybridized carbon lattice remains intact. Intermolecular interactions with materials exhibiting a π system are feasible [75, 77–79]. Especially porphyrins are promising, as they exhibit a metallic core, which can mimic enzymatic-like reactions or induce a change in the physical properties of graphene resulting in an opened bandgap, potentially tuning its intrinsic characteristics [80, 81]. One has to be cautious as there is no bandgap control upon non-covalent functionalization, which might be used in maximizing the detection sensitivity by minimizing the electrical noise [76]. Pyrene derivatives have been reported as non-covalent linker between graphene and other (bio-)molecules, which act either as probe or as target [32, 82, 83]. The π-interaction of graphene with carbon nanotubes enables the processability of the former hydrophobic carbon wires in aqueous solution [84]. The carbon surface is decorated by deposition of metallic nanoparticles, i.e., noble metals. Preferably, defective graphene materials are used as support, as it provides numerous nucleation sites for the deposition of the metallic nucleus growing to a particle [30, 35, 84–87]. Covalent modifications were initiated by anodization of graphene layers to introduce more oxygen functionalities or by a radical reaction, forming a diazonium salt that attacks the sp^2^-hybridized carbon lattice [71, 88]. Oxygen functionalities present at the surface of defective graphene compounds offer binding sites for linkage via carbodiimide coupling [77]. The covalent attempt is often found for biomolecular modified graphene compounds, as the amide bonds combine with the oxygen residues of graphene. Ultra-high pressures allow to tune the doping ratio of graphene surfaces [89]. The reactivity towards oxygen was shown to be enhanced by charge-doping under photothermal heating applying either positive or negative pulsed back gate voltages causing either electron doping or hole doping. Back gating without photothermal treatment did not result in a doping effect. The charge-doped-increased reactivity poses as an alternative to chemical-based catalysis, which can potentially enhance and control the chemical reactivity of a material [90]. A non-destructive functionalization technique is the creation of stacked van der Waals heterostructures. Structural relatives of two-dimensional graphene, e.g., transition metal dichalcogenides or hexagonal boron nitride, act as building blocks to tremendous possibilities of material design tuning the physical properties of the materialistic compounds [91, 92].

## Sensors

The direct interaction of graphene-like materials with analytes or ease of functionalization as well as the tenability to obtain materials either of metallic, semiconducting, or insulating character is highly attractive for electrochemical sensors. Excellent reviews focus on the sensing capabilities of 2D carbon materials towards numerous analytes [93–97]. A critical evaluation of the effect and requirements for 2D carbon materials in different types of electrochemical sensors is missing so far and presented in the following.

## Field-effect transistors and chemiresistors

**Fig. 7:**
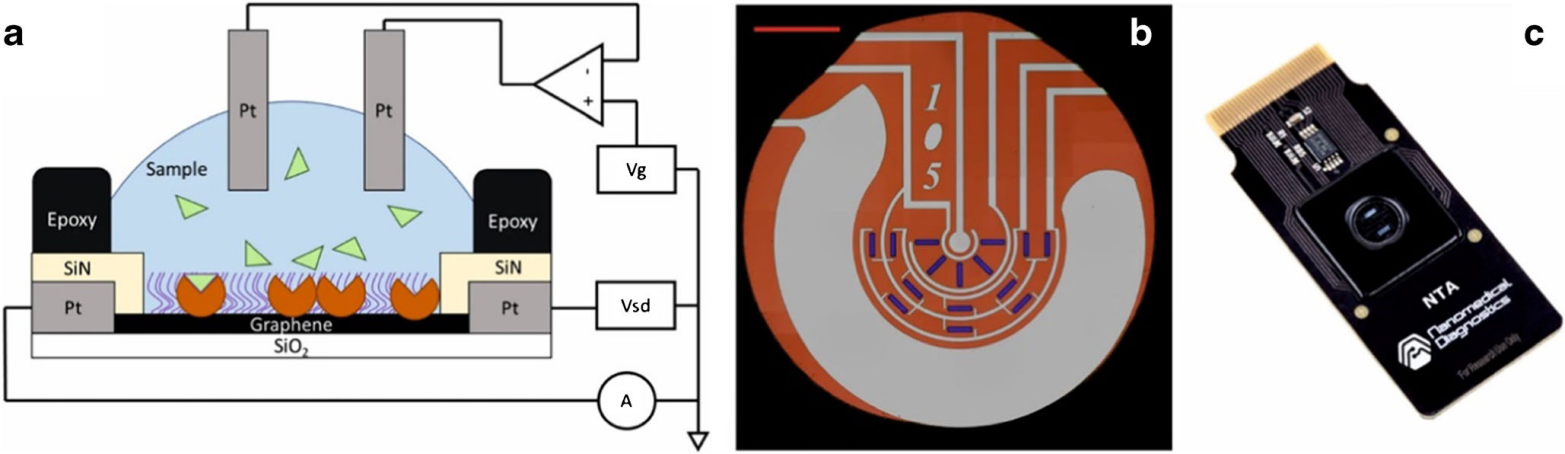
Scheme of a graphene field effect transistor with symbolized proteins linked to the 2D carbon material (**a**). The entire sensor surface of a commercial sensor showing five sensing spots with a red scale bar of 1 mm is depicted in (**b**). A photograph of the fully integrated graphene-based senor chip from Nanomedical Diagnostics is shown in (**c**). Adapted from [98]. Copyright (2019) Springer Nature

In field-effect transistors (FET), semiconducting 2D carbon materials act as channel between source and drain electrode applying a potential of the gate electrode. Two sensing types are accomplished, either back-gating or top-gating, also known as solution-gating. The interaction of the analyte with the 2D carbon material changes its charge carrier density due to electric charge distribution making this technology capable to develop rapid, miniaturized sensors [98]. A chemiresistor is similar to the FET. The detection principle remains the same, but it omits the gate electrode. The slightly more complex electronics of FETs compared to chemiresistors enable better sensitivity by tuning the conductance of the material by controlling the gate voltage. Both techniques can easily be adapted for commercialization. 2D carbon nanomaterials are preferably used due to excellent chemical stability and electric field sensitivity (Fig. 7) [98].

In a graphene FET (GFET), the material is accessible to surface functionalization as the channel is partially integrated into the device and the electrical double layer allows operation at low gate voltage with high conductivity. Monolayer graphene may be superior compared to multi-layered graphene as the conductance is improved. This is demonstrated by a DNA sensor, where the probe DNA was coupled to monolayer graphene via a pyrene butyric *N*-hydroxysuccinimide ester showing an outstanding limit of detection (LOD) of 25 aM for 24 mer target DNA [82]. A FET targeting at DNA, based on multi-layer graphene, was found to detect a 42 mer target DNA down to 10 fM. Here, graphene exfoliated by ultrasonication in DMF was used as channel material. The fabrication of the transistor is rather complicated. The substrate needs to be modified by glutaraldehyde and LPE graphene was drop-coated on top of the surface. The formation of a continuous film of carbon 2D material required a two times repetition [99]. To enhance the surface area, rGO was drop-casted onto a substrate to be used as channel material, providing a rough carbon layer. A peptide nucleic acid (PNA)–DNA hybridization FET was accomplished, by attaching the PNA by π–interaction of a pyrene-linker to the rGO. At constant bias voltage (*V*_*ds*_ = 0.1 V) the highly selective sensor showed a LOD of 100 fM for a 22-mer DNA strand [32]. These three examples in DNA sensing based on FET transduction show that a lower number of defects and a lower number of layers achieve slightly better detection limits. The superior conductivity of the material outperforms the enlarged surface area by improved signal-to-noise ratios.

On the other hand, defects are reported to enhance the sensitivity. This was demonstrated for a glucose sensor by fabricating a graphene mesh via CVD on a copper foil where certain areas were blocked by silica spheres [100]. The edge defects can be used to link the enzyme glucose oxidase (GOx) to the carbon material improving the charge transfer. Compared to the same sensor with cvdG, the sensitivity was enhanced from − 0.37 to − 0.53 mV mM^−1^ for the graphene mesh. A similar effect was reported, when it was discovered that certain graphene-based pH sensors can exceed the theoretical maximum Nernst limit of 59 mV pH ^−1^ in sensitivity. This was also attributed to defects, which allow in addition to the electrostatic gating effect a direct transfer of the charge carriers to the 2D carbon material [101].

**Fig. 8:**
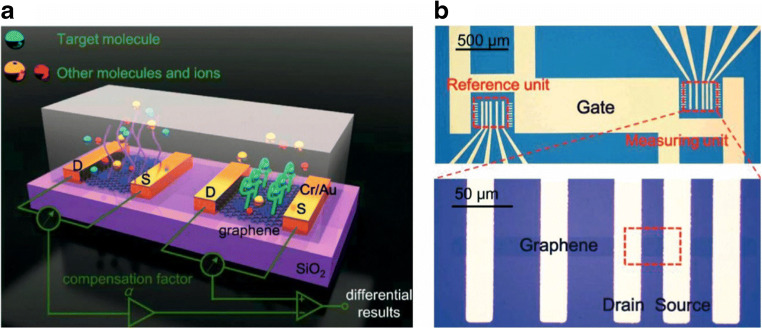
Scheme of a combination of two aptamer-modified GFETs allowing via differential measurement to distinguish between analyte binding and non-specific binding (**a**). Microscopic image of the electrode layout (**b**). Reprinted with permission from [103]. Copyright (2019) Elsevier

Disease-related small marker molecules like glucose [100], dopamine [87], urea [102], or environmental-related parameters such as 17β-estradiol [103], pH [104], heavy metal ions [105], or chlorine [106] have also be determined by GFETs. Selectivity can be achieved by using a differential measurement of two differently modified GFETs as it is illustrated in Fig. 8. This can easily be realized without complicating the fabrication process and due to the miniaturized size of GFETs, this does not affect the total size, the sample consumption, or the need of any complex electronics.

In general, cvdG is the material of choice in terms of miniaturization and mass production. Even when mass production still suffers from defects, originating of polymer contamination, affecting 44.2% of all chips [98], this material is easier to handle compared to carbon nanotubes or solvent dispersed carbon 2D materials in small dimensions. Transferred monolayers can be further etched by using masks in combination with oxygen plasma.

One of the rare exceptions of using rGO in FETs was reported by the group of Wolfgang Knoll. They report on a layer-by-layer (LbL) assembly of urease and polyethyleneimine on rGO and exploit the pH dependency of liquid-gated GFETs when detecting urea [102]. The LbL technique was chosen to neither disrupt the functionality of the biomolecule [107] nor to damage the sp^2^-hybridization of the graphene lattice [108]. Reduced GO is advantageous for pH sensing due to its defective structure, which allows a change in the surface charge density and the electric double layer when pH is changing, causing additional electrostatic gating effects [109].

**Fig. 9:**
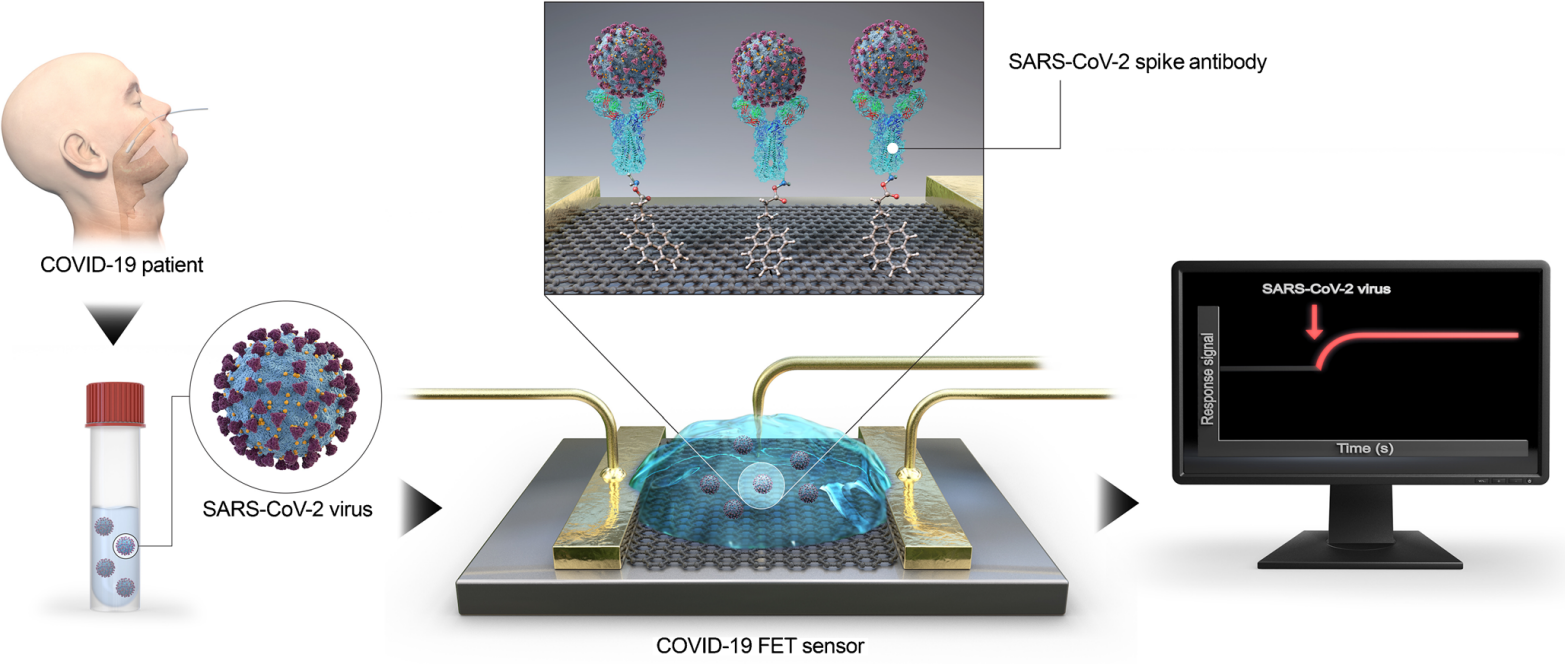
Schematic operation procedure of the COVID-19 FET using graphene as channel material, modified by 1-pyrenebutyric acid *N*-hydroxysuccinimide ester via π-stacking, coupling the SARS-CoV-2 spike antibody. Reprinted with permission from [110]. Copyright (2020) American Chemical Society

Graphene FETs have also been reported as a platform for immunosensors, e.g., to determine emerging pathogens such as coronavirus 2 (SARS-CoV-2) (Fig. 9). Graphene, as supporting layer, equips the FET with a highly conductive material providing a large surface area to enable low-noise detection of the virus. A SARS-CoV-2 spike antibody modified with a pyrene linker was assembled to cvdG by π-stacking. The sensor was highly sensitive for SARS-CoV-2 in clinical samples as well as selective compared to Middle East respiratory syndrome coronavirus (MERS-CoV). The detection limit is 1 fg mL^−1^ for the SARS-CoV-2 antigen protein. The sensor proved to be successfully applied in clinical diagnosis as nasopharyngeal swab specimen from COVID19 patients and healthy persons were investigated [110].

**Fig. 10:**
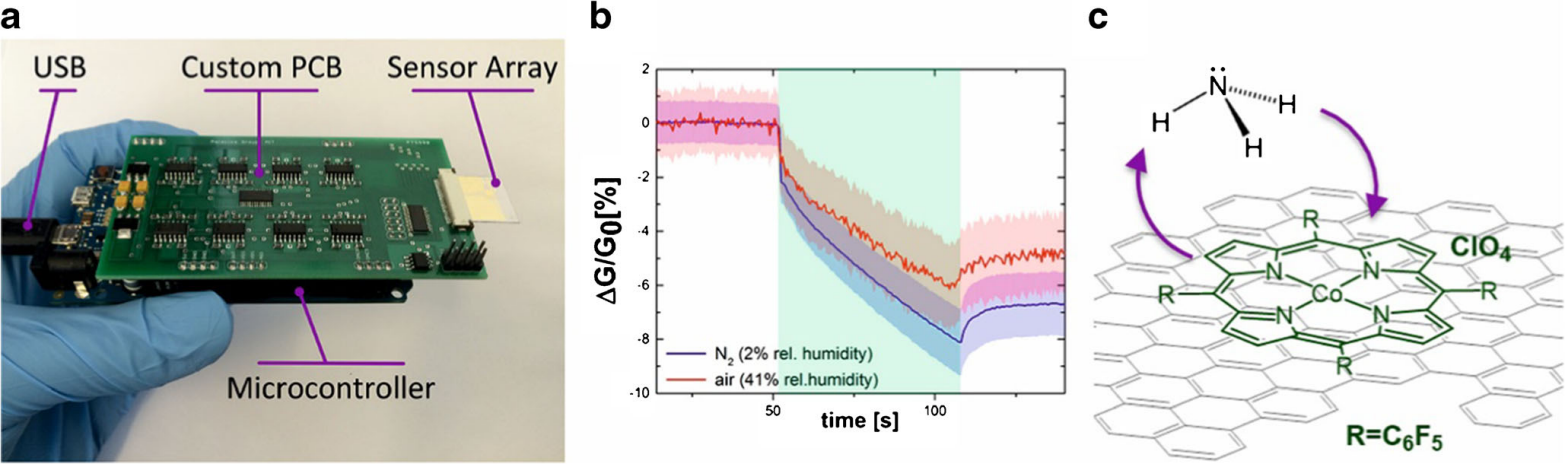
A measurement device with implemented sensor array (**a**), recording the change of conductivity upon exposure to gaseous NH_3_ under varying environmental conditions (**b**). The sensitivity is enhanced by graphene functionalized with cobalt meso-arylporphyrins, which interact with the analyte (**c**). Reprinted with permission from [111]. Copyright (2018) American Chemical Society

Chemiresistors are extremely popular in gas sensing. Here, 2D carbon materials can add additional value by their high affinity to gasses, together with excellent conductivity and catalytic effects. Figure 10 shows a measurement device with an array of chemiresistors recording the change of conductance in presence of NH_3 gas_ under different environmental conditions, which is enhanced by the recognition elements, cobalt meso-arylporphyrins [111].

The high operating temperatures (often > 200 °C) are one of the major drawbacks in gas sensing with semiconducting metal oxides, which can be overcome. Ammonia is one of the most frequently studied analytes, as it is harmful to humans already at very low concentrations. The threshold concentration is 25 ppm for an exposure time of 8 h [112]. Besides safety issues, ammonia is also known as health marker in breath or to monitor food quality [113]. Graphene oxide reacts to ammonia with functional groups, accompanied by a decrease in its conductivity, which is usually attributed to p-doping of physically adsorbed oxygen. To introduce selectivity, several functionalization strategies have been studied. The most promising are phthalocyanines, especially with Cu as central metal, to be used in breath analysis [114]. A high selectivity was found for many volatile organic compounds (VOCs) and what is more, the combination of GO with Cu tetra-β-amine-phthalocyanines strongly reduces the cross-sensitivity to humidity. Humidity is one of the major interfering substances when it comes to the quantification of gases at room temperature. For low relative humidity (r.h.) (< 30%), water adsorbs on the 2D material’s surface and acts as an electron acceptor, which increases the electrical conductivity. When r.h. gets larger, the water molecules adsorbed on the sensor surface can be ionized, H_3_O^+^ is formed, which acts as charge carrier. In such cases, both electrical and ionic conductivity will be enhanced and the sensor is interfered. Therefore, the choice of the 2D carbon material has an impact on sensitivity as well as on selectivity. The complexity in the surface chemistry of 2D materials decreases in the order of GO < rGO < LPE graphene < cvdG. In the same order, the conductivity of the material increases. A large variety in surface chemistry might be attributed to many binding sites, either for adsorption of the gas itself or for easy modification with metal or metal oxide nanoparticles, which further increase the surface area, number of binding sites, binding affinities, or even may introduce electrocatalytic effects. Table 1 summarizes the recent achievement of ammonia sensors operated at room temperature in the order of the carbon materials used. A remarkable short response and recovery time of few seconds only was found when using rGO in combination with silver nanoparticles [115] or SnO_2_ nanorods [116]. This might be ascribed to the catalytic effects of Ag_2_O and the SnO_2_ surface of the particles/rods with generated radicals, which are known for a fast reaction. In comparison of silver-nanoparticle-modified 2D carbon materials, a huge difference in the range of detection is found for cvdG [117] and rGO [115]. This can be attributed to the smaller metallic particles (50 nm in size) on rGO compared to 200-nm sized particles on cvdG. A reason for the bigger nanocrystals on the less defective carbon 2D material could be that defects act as nucleation sites, which allow better control of the reduction step. An outstanding low LOD in the ppt range was achieved by a vertical alignment of cvdG [58]. The authors highly attributed the hydrophobicity (water contact angle: 137°), the ultra-high specific surface area, exposed sharp edges, and the unique non-stacking three-dimensional geometry to be beneficial in reaching such high sensitivity. The drawback of this method is to be found in the device fabrication, which is rather time-consuming. For other gaseous analytes, the choice of the 2D material and the functionalization strategies can easily be derived from the examples shown for ammonia.

**Table 1:** Chemiresistive transducers based on differently fabricated 2D carbon materials for quantification and determination of ammonia at room temperature

Material	Modification	LOD	Range/ppm	Response/recovery	r.h./%	Selectivity	Comment	Reference
cvdG	–	–	(0.1–1) 10^−4^	4 min/few min (−)	–	–	Vertically aligned cvdG	[58]
cvdG	Co *meso*-arylporphyrins	–	20–160	60 s/− (−)	41	VOCs	FET	[111]
cvdG	Ag NPs	–	(0.05–1.2) 10^4^	120 s/70 s (−)	30	VOCs	–	[117]
ecG	ZnO nanowires	–	0.5–50	6 s/36 s (0.5 ppm)	–	VOCs, benzene, nitrobenzene, nitrotoluene	–	[118]
rGO	Ag NPs	1.2 ppb	0.1–15	5 s/6 s (0.1 ppm)	25	H_2_, CO	–	[115]
rGO	In_2_O_3_ nanofibers	44 ppb	1–60	17 s/214 s (−)	30	Organic solvents, nitrogenated compounds	Impedance at 10 kHz	[119]
rGO	Mesoporous PPy layer	200 ppb	10–40	5 min/10 min (−)	–	VOCs, acetonitrile, chlorobenzene, EA, toluene	No influence on humidity	[120]
rGO	MXene	–	10–50	–	–	VOCs, H_2_S, SO_2_, xylene, benzene	–	[121]
rGO	SnO_2_ nanorods	20 ppm	20–3000	8 s/13 s (200 ppm)	45	VOCs, NO, CO, H_2_S	No influence on humidity	[116]
GO	Fluorinated	6 ppb	0.1–0.5	86 s/116 s(−)	–	VOCs, NO_2_, H_2_, toluene	–	[122]
GO	Cu tetra-β-amine-phthalocyanine	–	0.8	−/350 s(−)	–	VOCs, NO_2_	No influence on humidity	[114]

*PPy* polypyrrole, *EA* ethyl acetate

## Impedimetric, amperometric, voltammetric sensors

Graphene-modified electrodes as impedimetric sensors are popular in determination of biomolecules, biomarker, proteins, or DNA. Mostly, the sensing mechanism depends on the blocking of the electrode upon analyte binding, which inhibits the interaction of the electrode with a redox marker present in the electrolyte. Table 2 presents graphene-based sensors to determine proteins or clinically relevant biomarkers by electrochemical impedance spectroscopy.

**Table 2:** Selection of various graphene-modified impedimetric sensors to quantify proteins and disease-relevant biomarkers

Material	Modification	Analyte	Linear Range	LOD	Selectivity	Reference
rGO	MWCNT-Au NPs-chitosan	Lysozyme	0.02–250 pM	9 fM	BSA, HSA, Hb, thrombin, IgG, CytC, PSA, enzymes	[123]
rGO	MWCNT-Au NPs	PSA	5–0.1 μg mL^−1^	1 pg mL^−1^	BSA, Hb, thrombin, IgG, lysozyme	[124]
rGO	SWCNT-Au NPs-aptamer	HER2	0.1 pg mL^−1^ - 1 ng mL^−1^	50 fg·mL^−1^	BSA, BHb, thrombin, IgG, lysozyme, PSA	[125]
cvdG	Anti-OVA	Ovalbumin	1–0.1 pg mL^−1^	0.9 pg mL^−1^	β-LG, lysozyme	[88]
cvdG	Anti-CEA	CEA	1.0–25.0 ng mL^−1^	0.23 ng mL^−1^	KCl, CYFRA-21-1, CTnI	[83]
cvdG	Anti-IgG	IgG	0.1–100 μg mL^−1^	0.136 μg mL^−1^	BSA, Hb, avidin	[78]

*BSA* bovine serum albumin, *CEA* carcinoembryonic antigen, *(MW/SW)CNT* multi-walled/single-walled carbon nanotubes, *CRP* c reactive protein, *CTnI* cardiac troponin I, *CytC* cytochrome C, *(B)Hb* (bovine) hemoglobin, *HER2* human epidermal growth factor, *HSA* human serum albumin, *β-LG* β-lactoglobulin, *Mb* myoglobin, *PSA* prostate-specific antigen

**Fig. 11:**
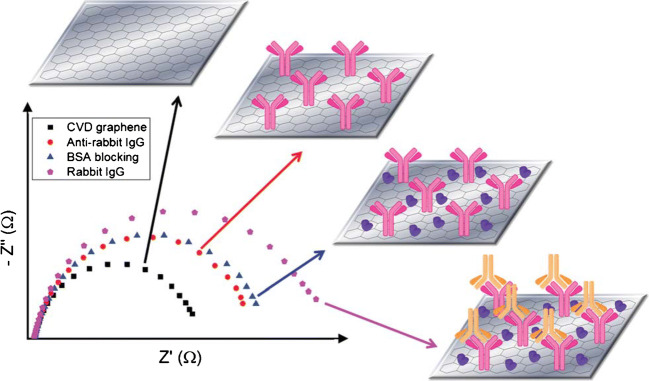
Impedance behavior in the presence of a redox marker for cvdG (black), anti-IgG/cvdG (red), BSA/anti-IgG/cvdG (blue), and IgG/BSA/anti-IgG/cvdG (pink), respectively. Reproduced from [78] by permission of The Royal Chemical Society

An impedimetric immunosensor was established by using cvdG. The protocol forsakes a physical adsorption of the antibody towards rabbit immunoglobulin G (IgG) onto a multi-layered cvdG, grown on nickel [78]. Edges and wrinkles became present within the layered carbon material. The physical adsorption of anti-IgG is followed by a blocking step with BSA. Compared to the introduction of linker molecules, the non-destructive physical adsorption appeared to be superior as it leaves the sp^2^-hybridized structure of graphene intact, retaining the physical properties. Electrochemical impedance spectroscopy revealed an increase in the charge transfer resistance (R_CT_) from bare cvdG via an anti-IgG/cvdG to a BSA/anti-IgG/cvdG assembly (Fig. 11). The incubation with rabbit-IgG led to an even more enhanced R_CT_, capable to determine the analyte with a LOD of 0.136 μg mL^−1^ IgG. Only after 7 days of storage, the signal response of this sensor drops to 48%. This demonstrates a drawback of the physical adsorption of biomolecules in receptor design on 2D carbon materials.

Better stability can be achieved by attaching a receptor covalently. This is difficult for cvdG as it exhibits only functionalities upon an imperfect handling. A straightforward approach was developed by electrografting of 4-aminobenzoic acid on cvdG to design a sensor for the determination of ovalbumin (OVA) [88]. Even though cvdG was supposed to convince with the exceptional electrical properties of a low-defective material, it was not taken into consideration that a covalent functionalization routine destructs the sp^2^-hybridized carbon lattice, changing the electronic properties of the material, i.e., by opening a bandgap. The LOD was 0.9 pg mL^−1^ OVA in a linear range of 1 pg mL^−1^ to 100 ng mL^−1^.

**Fig. 12:**
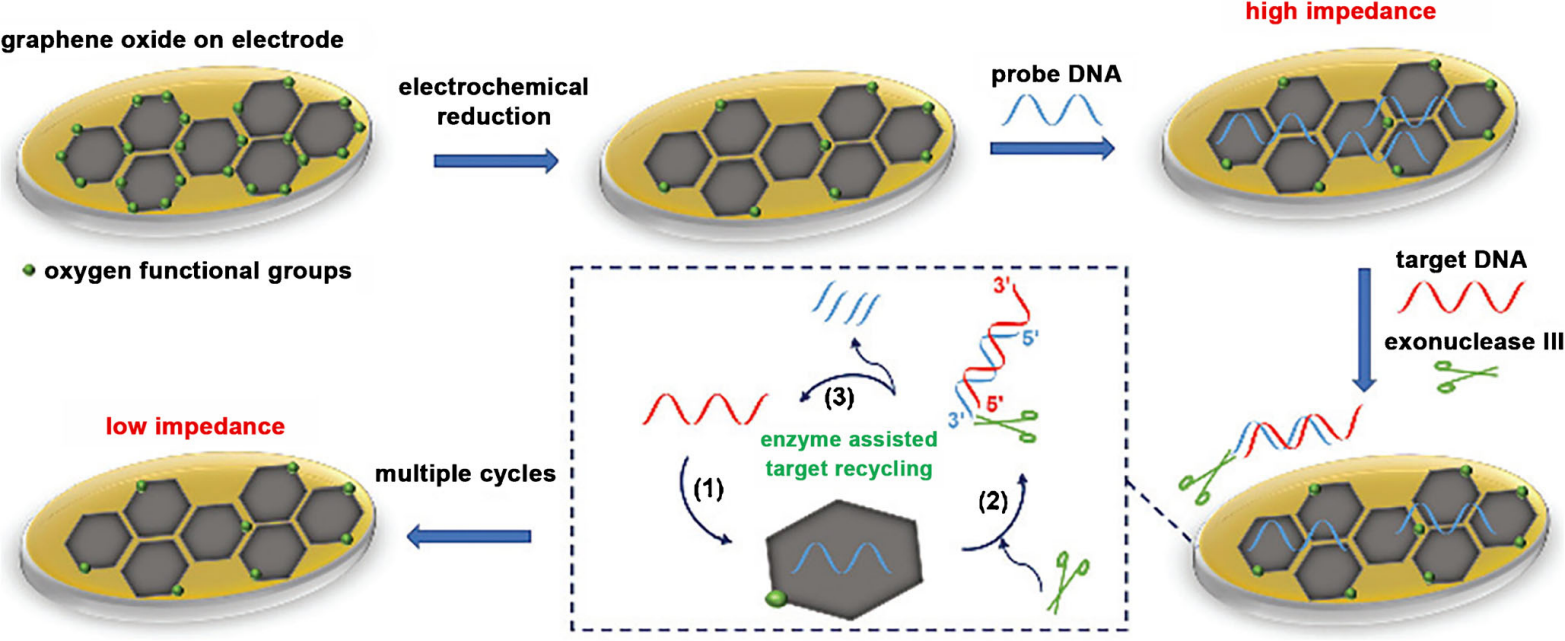
Scheme of an impedimetric sensor based on rGO, which is modified by adsorption of probe DNA. The determination of complementary target DNA is accompanied by enzyme-assisted sensor recycling. Reproduced from [79] by permission of The Royal Society of Chemistry

A comparison of electrochemically reduced graphene oxide and untreated graphene oxide was drawn to investigate the ability to improve the sensing performance towards DNA upon label-free electrical and enzymatic signal amplification [79]. The non-covalent modification of graphene layers with probe DNA increases the R_CT_ value between redox marker and electrode surface due to electrostatic repulsion. Upon hybridization with the target DNA, the resistance increases even more. The enzyme exonuclease III recycles the sensor layer upon cleavage of the dsDNA (Fig. 12).

Investigations on DNA with various concentrations were performed for either GO modified electrodes (10 fM–1 nM ssDNA) or rGO modified electrodes (5 aM–1 nM ssDNA). The GO-modified electrode revealed a LOD of 50 fM DNA, whereas the LOD is significantly decreased to 10 aM for rGO modified electrodes. Compared to GO which is almost an insulating material due to the enormous structural irregularities, highly conductive rGO is sensitive towards surface processes resulting in an enhanced change of R_CT_ upon blocking and regeneration of the electrode material. A regenerated sensor layer did not suffer a loss of performance, whereas it became apparent that upon increased incubation time of the enzyme, artifacts regarding matrix adsorption as well as spontaneous enzyme deactivation might take place [79].

In contrast to glassy carbon (GC), rGO provides a high affinity to aromatic molecules as demonstrated by porphyrins, which easily attach by π-stacking to rGO. A metallic porphyrin-modified rGO impedimetric sensor was prepared to capture hepatitis C-DNA by a complementary ssDNA, which is linked to manganese(III) tetraphenylporphyrin (MnTPP) [80]. The non-covalent modification left the sp^2^-hybridized carbon lattice intact, maintaining high conductivity. In contrast to pyrene groups, porphyrins are able to incorporate an electrochemically active metal ion. The manganese ion served to monitor the stability of the porphyrin linker attached to the electrode during electrochemical measurements. The use of rGO as supporting material prevented the MnTPP from detaching, in contrast to a glassy carbon electrode (GCE). An increased electron transfer attributed to graphene’s high surface area as well as the short distance between graphene and porphyrin result in a sufficient sensor performance [80].

Table 3 presents impedimetric graphene-modified sensors that determine DNA strands, potentially applicable in clinical diagnosis to identify viruses, bacteria, as well as relevant biomarkers. Many allotropes from the pool of graphene materials can be found as sensor material, but the reduced graphene oxide is the most frequently used compound, due to the well-established fabrication protocols. The functional groups present at the material’s surface enable easy functionalization by deposition of conductive polymers [126, 127], liposomes [128], metallic nanoparticles [129–132], or porphyrins [80] to pave the way for enhanced analysis strategies.

**Table 3:** Impedimetric graphene–modified sensors to determine ssDNA. Every sensor layer is additionally modified with a specific capture probe DNA

Material	Modification	Nucleotides	Linear range	LOD	Selectivity	Reference
GO	Chitosan	21	10 fM–10 nM	3.6 fM	Mismatched	[133]
carboxylated graphene-nanoflakes	APTMS-ZnO	17	0.1 fM–1 μM	0.1 fM	Mismatched, non-complementary	[134]
rGO	Exonuclease III	23	50 aM–1 nM	10.0 aM	Mismatched	[79]
rGO	Liposome	24	1 μM–1 fM	10.0 aM	Mismatched, non-complementary	[128]
rGO	Au NPs-PABA	20	0.1 fM–10 nM	37 aM	Mismatched	[129]
rGO	PANI	23	0.5 fM–0.1 nM	0.1 fM	Mismatched, non-complementary	[126]
rGO	PPy-CO_2_H	19	10 fM–10 nM	3 fM	Mismatched, non-complementary	[127]
rGO	ZrO_2_-hairpin probe DNA	22	10 fM–0.1 nM	4.3 fM	Mismatched, non-complementary	[135]
rGO	Nafion	–	0.1 pM–0.1 nM	23 fM	Mismatched	[136]
rGO	Au NPs	30	0.1 pM–0.1 μM	36 fM	Mismatched, non-complementary	[130]
rGO	MnTPP	25	0.1 fM–10 pM	61 fM	Mismatched, non-complementary	[80]
rGO	Au NPs	18	10 nM–20 μM	0.18 nM	Non-complementary	[131]
cvdG	oxygenated	15	2 aM–1 pM	1.0 aM	Mismatched, non-complementary	[137]
ecG	–	–	0.2–5 pg mL^−1^	0.68 pg mL^−1^	–	[138]
Ultrasound-exfoliated graphene	ZnO	25	10 pM–1 μM	4.3 pM	Mismatched, non-complementary	[132]

*APTMS* (3-aminopropyl)trimethoxysilane, *PABA* 4-aminobenzoic acid, *PANI* polyaniline (emeraldine salt)

In contrast to frequency-dependent impedance spectroscopy, which monitors binding events in an equilibrium, time-dependent sensing techniques, e.g., voltammetry and amperometry, are attractive to investigate induced electrochemical reactions.

Numerous exfoliation protocols strive for the preparation of graphene materials with a low number of defects to come close to the ideal graphene. For time-dependent electrochemical sensing techniques, the presence and number of defects within the graphene affects their electrochemical activity. The defect-dependent sensing capability of the 2D carbon compounds generated by sonication was investigated upon the determination of phenolic compounds. The quality of the carbon materials ranges from a low amount of defects present within the structure to graphene compounds, which were anodized to provide additional functional groups [71]. NMP-dispersed-graphite powder was exfoliated in a sonication bath (40 kHz, 100 W) for 48 h yielding graphene sheets. For a comparative study, GO was synthesized by a modified Hummers’ method. Glassy carbon electrodes were modified by drop-casting of graphene and drying under an infrared lamp. The LPE graphene-modified electrodes were anodized (1.8 V for 2 min) in acetate buffer (pH 5.6). Atomic force microscopy revealed the roughest surface for sonication-derived graphene-modified GCE, potentially exhibiting the highest number of active sites. Differential pulse voltammetry (DPV) for all phenolic compounds revealed none or very weak oxidation peaks for GCE and GO-modified electrodes. The oxidation peaks are enhanced and shifted to more negative potentials for graphene/GCE and anodized graphene/GCE. The LODs are calculated to be 12 nM, 15 nM, 10 nM, and 40 nM for hydroquinone, catechol, 4-chlorophenol, and 4-nitrophenol, respectively [71]. The electrochemical sensor performance of differently prepared graphene compounds was examined for a wide range of electrochemically active analytes like biomolecules, synthetic colorants, and phenolic compounds (4-chlorophenol, 4-nitrophenol)) using DPV. The plain GCE was not able to determine the analytes at low concentrations, but as the amount of defects increased within the graphene materials, the oxidation current of the different analytes increases [70]. Although the addition of salts might enhance the exfoliation efficiency of graphene via LPE, one of the advantages of fabrication by ultrasound is the possibility to prepare 2D materials in appropriate medium without the need of any additives, potentially influencing the physical behavior of the material. Comparing the detection limits of salt-assisted exfoliated graphene to sonicated graphene subsequently anodized, the LOD for 4-chlorophenol and 4-nitrophenol was increased by a factor of two and of six, respectively. The extended sonication time seems to be a promising alternative to prepare graphene compounds, instead of using additives, potentially influencing the material.

Electrochemical graphite exfoliation is predestined for simplification as the preparation procedure and material functionalization can be combined to one step, which was proved for a graphene-porphyrin compound. Graphite was exfoliated electrochemically with porphyrin as intercalant, simultaneously functionalizing graphene flakes by π-interaction, introducing an oxidizing agent for catechol [81]. Apparently, the material exfoliated by means of additional salts weakens the performance of the established electrochemical sensor with regard to liquid-phase exfoliation of graphene derived solely by exfoliation in appropriate solvent. The generation of 2D-based heterostructures incorporating catalytically active compounds is required to improve the performance of sensors towards analyte determination.

The design of improved electrochemical sensors envisions the hybridization of electrocatalytic active groups with graphene substrates. Besides the functionalization of graphene with carbon allotropes by π-stacking, highly attractive became the decoration with electrocatalytic active metallic nanoparticles (NPs). They provide a suitable substitute to natural enzymes, as they are more stable and are less prone to operation and environmental conditions. Metallic nanoparticles combine easily with carbon materials upon hydrothermal route or by direct electrodeposition onto the substrate of choice [86, 124, 125, 129, 132, 134, 139–143]. For successful analyte determination upon catalytic reaction, the achievement of homogeneously distributed small metallic NPs exhibiting a large electrical active surface is crucial. Nevertheless, it became apparent that deposited NPs tend to agglomerate on polycrystalline materials, i.e., GC due to inhomogeneous charge distribution. This drawback is targeted by using graphene as supporting material, which prevents the particles from agglomeration resulting in homogeneously distributed monodisperse NPs creating an electrochemical synergy of the carbon-metal-interface [144].

**Fig. 13:**
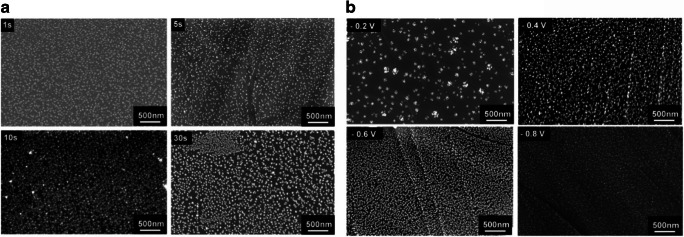
SEM images of AgNPs modified cvdG for 1 s, 5 s, 10 s, and 30 s in 0.2 M KNO_3_ containing 0.7 mM AgNO_3_ stepping the potential from 0 to − 0.4 V (**a**). AgNPs were deposited on cvdG at potentials of − 0.2 V, − 0.4 V, − 0.6 V, and − 0.8 V, respectively, for 30 s (**b**). All potentials are given vs. Ag/AgCl. Adapted from [141] with permission from The Royal Society of Chemistry

The diameter of nanoparticles is crucial towards the electrocatalytic behavior, as the potentials of redox reactions can be shifted by the nanoparticle’s size and shape. The size of the particles can be either modulated by applied deposition voltage or deposition time, as shown in Fig. 13.

Tables 4 and 5 provide an overview of graphene-modified electrodes targeting hydrogen peroxide or glucose, both frequently determined in bioanalysis. To enhance the sensitivity and selectivity, catalytically active metals replaced enzymes almost completely influencing the operation potential. An amperometric gold-GO-based sensor was prepared by simultaneous electrodeposition of GO containing HAuCl_4_, forming a AuNPs-modified rGO hybrid [86]. Gold nanoparticles were chosen as catalyst due to its stability and operation in neutral medium. An aqueous dispersion of GO was electrodeposited on gold electrodes by application of a potential of − 30 V for 20 min, reducing GO. The graphene support is necessary to stabilize AuNPs, which tend to agglomerate otherwise. Additionally, graphene enhances the glucose oxidation process. Amperometric measurements were performed in phosphate buffer (pH 7.4) at a potential of 0 V vs. Ag/AgCl, subsequently adding glucose with increasing concentration, exhibits a LOD of 12 μM.

**Table 4:** Amperometric sensors based on graphene-modified electrodes to determine glucose. All potentials were measured against Ag/AgCl when not stated otherwise

Material	Modification	Potential/V	Linear range	LOD/μM	Selectivity	Reference
LIG	Cu NPs	− 0.4 (SCE)	1–4.54 μM	0.35	AA, UA, DA, AP, Fru, Lac, Suc	[145]
N-rGO	Cu NPs	0.6	0.01–100 μM	0.01	AA, UA, DA, Fru, Lac, Suc	[146]
N-rGO	Ni(OH)_2_ nanorods	0.45	0.5–11.5 μM; 11.5–240 μM	0.12	AA, UA, DA, Fru, Lac, Suc	[147]
rGO	Cu NPs-PANI	0.5 (SCE)	1 μM–0.96 mM	0.27	AA, UA, DA, Lac, Suc	[148]
rGO	Cu nanoflower	0.6	2 μM–2 mM; 2–13 mM	0.5	AA, UA, DA, NaNO_3_	[143]
rGO	Ag-CuO NPs	0.6	0.01–28 mM	0.76	AA, UA, DA, Gal, Lac, Suc	[139]
rGO	Ni-doped MoS_2_ NPs	0.55	0.005–8.2 mM	2.7	AA, UA, DA, AP	[149]
rGO	PtAu NPs -GOx-chitosan	0.45	0.005–2.4 mM	5	AA, UA, AP	[142]
rGO	Au NPs	0	0.05–14 mM; 14–42 mM	12	AA, UA, DA, urea	[86]
CVD	Au NPs	0	6 μM–28.5 mM	1		[150]
oxygenated-CVD	GOx-Nafion	− 0.5	0.4–2 mM	124.2		[151]

*AA* ascorbic acid, *AP* 4-acetamidophenol, *DA* dopamine, *Fru* fructose, *Gal* galactose, *Lac* lactose, *LIG* laser-induced graphene, *PANI* polyaniline (emeraldine salt), *Suc* sucrose, *UA* uric acid

**Table 5:** Amperometric sensors based on graphene-modified electrodes to determine hydrogen peroxide. All potentials were measured against Ag/AgCl, when not stated otherwise

Material	Modification	Potential/V	Linear range	LOD/μM	Selectivity	Reference
LIG	Ag NPs	− 0.5	0.1–10 mM	7.9	AA, Glu,	[152]
3D-N-doped rGO	NiCo_2_O_4_ nanoflowers	0.5	1–510 μM	0.136	AA, UA, DA	[153]
rGO	nAPAMSs	− 0.5	0.005–4 mM	0.008	AA, UA, AP, Glu	[35]
rGO	Fe NPs	− 0.5	0.1 μM–2.15 mM	0.056	AA, UA, DA, CT, Glu	[154]
rGO	Ferumoxytol-Pt NPs	0.1	0.4–10 μM; 0.0075–4.3 mM; 4.9–10.8 mM	0.38	AA, DA, Cys, GSH, Fru	[155]
rGO	Cu_2_O-PANI	− 0.2 (SCE)	0.8 μM–12.78 mM	0.5	AA, UA, Glu	[140]
rGO	Nafion-Ag NPs	− 0.6 (SCE)	1–30 mM	0.54	AA, UA, DA, Glu, urea	[30]
CVD	Au NPs	0.5	25 nM–1.5 mM	0.01	–	[156]
CVD	Pd NPs	− 0.12	4 μM–13.5 mM	1.5	–	[157]
CVD	Fe_3_O_4_ NPs	0.7	12.5–112.5 μM	4.4	–	[158]
Sono-ecG	–	− 0.4	0.02–2.9 mM	2.67	AA, UA	[159]

*AA* ascorbic acid, *AP* 4-acetamidophenol, *Cys* cysteine, *DA* dopamine, *Fru* fructose, *GOx* glucose oxidase, *GSH* glutathione, *Lac* lactose, *LIG* laser-induced graphene, *nAPAMS* nano Au and Pt alloy microsphere, *PANI* polyaniline (emeraldine salt), *Suc* sucrose, *UA* uric acid

A ternary hybrid composed of graphene, AgNPs and Nafion, targets the determination of H_2_O_2_ [30]. The combination of material enhances the surface area, the conductivity, and leads to stable compounds. Silver pose electrocatalytic activity but tend to oxidation, which requires the protective Nafion layer. The hydrothermal synthesis of the heterostructure was accomplished by a one-pot approach, mixing the precursor materials, GO dispersion, Nafion solution, and AgNO_3_. Glassy carbon electrodes were modified by drop-casting of rGO-Nafion-AgNPs. Cyclic voltammetric studies showed that an excessive AgNPs decoration led to the deterioration of the reduction ability of the sensor, as the aggregation of AgNPs reduces the electrocatalytic active sites of the material. Amperometric sensing was performed by applying a potential of − 0.65 V revealing a LOD of 0.53 μM H_2_O_2_, with a high selectivity for H_2_O_2_ compared to the five times increased concentration of urea, glucose, dopamine, uric acid, and ascorbic acid [30]. In contrast, a H_2_O_2_ sensor was prepared consisting of a heterostructure, decorated by microspheres of noble metals, Au and Pt, supported by rGO [35]. Reduced graphene oxide convinced as support due to higher conductivity and better stability. Furthermore, the lattice defects and functional groups pose attractive sites for deposition of metallic particles, which prevent them from aggregation. The solutions of GO, H_2_PtCl_6_, and HAuCl_4_ were mixed in equal molar ratio adding NaBH_4_ to trigger the reduction reaction. A GC electrode was drop-coated with rGO/Au/Pt. To detect H_2_O_2_ amperometrically, a potential of 80 mV vs. Ag/AgCl was applied, subsequently injecting the analyte into PBS (pH 7) resulting in a low LOD of 0.008 μM [35]. Apparently, even though noble metals are more expensive, they provide an enhanced electrocatalytic behavior in combination with graphene due to synergistic effects.

## Conclusion

Two-dimensional carbon materials have been intensively studied regarding synthesis, fabrication, and their implementation in electrochemical sensors. The fact that this class of nanomaterial is much more than a sp^2^-hybridized honeycomb-lattice of carbon atoms becomes vivid, when investigating the physical and chemical properties of the material, depending on the exfoliation technique. It can be stated that the preparation method guides the performance of the sensor material due the different intrinsic characteristics.

The variances originate from defects distributed within the carbon lattice, layer inconsistencies, oxygen functional groups, and adatoms. The still ongoing development of fabrication methods for 2D carbon materials in the last years’ aims for a high-yield production with a reproducible quality. Overcoming the weak interlayer van-der-Waals forces results in the delamination of the bulk material, yielding robust and flexible nanomaterial flakes due to strong intralayer forces [40, 160].

In 2018, the group of Castro-Neto has investigated the quality of graphene supplied from 60 different producers with the result that the quality of these materials is rather poor, and this is claimed as one of the key issues for the slow development of applications [161]. Especially for liquid-dispersed graphene, it is difficult to produce these batches with high reproducibility in quality. There is also information missing on the aging of such dispersions restacking of the material. In contrast to dispersions, cvdG shows better reproducibility. It is expected that challenges in reproducibility can be overcome by better characterization techniques [161].

The modification of the 2D materials are easily achieved either by covalent or non-covalent functionalization to improve the selectivity of the sensor [75, 76]. On the one hand, covalent modifications are reported to damage the sp^2^-hybridized carbon system of graphene. On the other hand, the linkage of active biomolecules might become an issue as the natural structure of the biomolecule can be disrupted followed by a loss of activity [102].

Numerous sensors were reported for an enormous variety of analytes based on 2D materials beyond the scope of this review. Different exfoliation techniques yield 2D carbon materials of a diverse structure, which consequently exhibits altered physical properties. Graphene, derived by CVD, is rather integrated in FETs or chemiresistors, as these sensors base on the change of the intrinsic electrical characteristics of the sensing layer upon analyte adsorption. The advantage of this material is claimed to be the intact sp^2^-hybridized carbon lattice, but for most electrochemical sensors, this is not a limitation so far. It is to point out that cvdG is prone to an unintentional introduction of non-reproducible defects, i.e., smallest variations of the processing parameters, complex transfer protocols inducing ionic impurities, and structural defects, deteriorating the physical and chemical properties. Besides the rather high-quality cvdG, synthetically derived rGO is widest applied as sensor layer since it is either produced in an ordinary laboratory or purchased from the growing market of suppliers offering graphene materials up to a liter-range. A large defect density is distributed along the material’s basal planes. Even though this term has a negative connotation, those structural defects do not pose a drawback when it comes to sensing applications. Quite the contrary, the defects are resembled as active functional groups, preferably interacting with a potential analyte, when applied as sensor surface. Not any other material was used as frequently as reduced graphene oxide in sensor design, due to the simplicity of the material and its physicochemical properties. Additionally, the oxygen moieties enable the dispersibility of rGO in water.

In terms of easily derived, low-cost, liquid processable material, one has to keep in mind the LPE-process of graphite. The exfoliation via sonication or shear exfoliation is widely performed either in toxic, high-boiling point, organic solvents with a matching surface tension to the material or in aqueous solution with a stabilizing surfactant. Two-dimensional materials are obtained without a chemical change in the basal plane of their structure, but with varying flake size and number of layers from mono- to multi-layered compounds. The diverse surface chemistry often affects the physical properties in a non-reproducible way. The mechanical exfoliation process may not introduce structural irregularities within the carbon layer, yet the number of defects increase upon formation of smaller flakes dominated by edge-planes. Electrochemical exfoliation is performed in aqueous solution using inorganic salts as intercalating species upon application of a high voltage. A bonus is the possibility of direct functionalization during exfoliation. Many reports exist on how to exfoliate 2D materials, but little is derived on the sensing behavior, especially when the morphology of the materials changes upon variation of the delamination process. Nevertheless, the reported LPE-derived materials applied in impedimetric or amperometric sensors revealed promising results. Future trends envision the use of all carbon-fabricated wearable electrochemical sensors. For a direct application on the skin, deeper understanding of the toxicity of each class of 2D carbon materials is still needed. The sustainability of the preparation procedures is barely addressed even though the growing demand of production and processing procedures result in an enhanced consumption of resources.

A deeper understanding of the electrochemical properties of the 2D carbon materials has to be obtained to unravel the question, whether a graphene material suits the requirements of the potential sensor. As stated before, the widest applied materials for sensor development were contrary materials, cvdG and rGO, known for either high-quality or defective graphene. Instead of implementing the materials according to their unique features, it was found that the intact sp^2^-hybridized carbon system of cvdG was intentionally destructed by introduction of sp^3^-hybridized functionalities. To become an essential part in mass-produced electrochemical sensors, the system integration must be compatible to already existing complementary metal-oxide-semiconductor (CMOS) technologies [162]. It is vital to understand which material will meet the sensor’s need to improve its performance. The electrochemical features have to be explored and further correlated to the chemical structure of the material. The obtained fundamental knowledge of physical and chemical correlations might be the key to tune the material’s properties directly to the special requirements, which would lead to much smarter sensors in the future.
